# Supplementary material for: ctDNA and tumor-based biomarkers of giredestrant response in acelERA breast cancer
Source: Nat Commun. 2026 Mar 12;17:3848. doi: 10.1038/s41467-026-70335-0 (PMC13121440; doi:10.1038/s41467-026-70335-0)
Supplement: Supplementary file 1 — Supplementary information [file 41467_2026_70335_MOESM1_ESM.pdf]

Supplementary Information

"ctDNA and tumor-based biomarkers of giredestrant response in acelERA Breast Cancer"

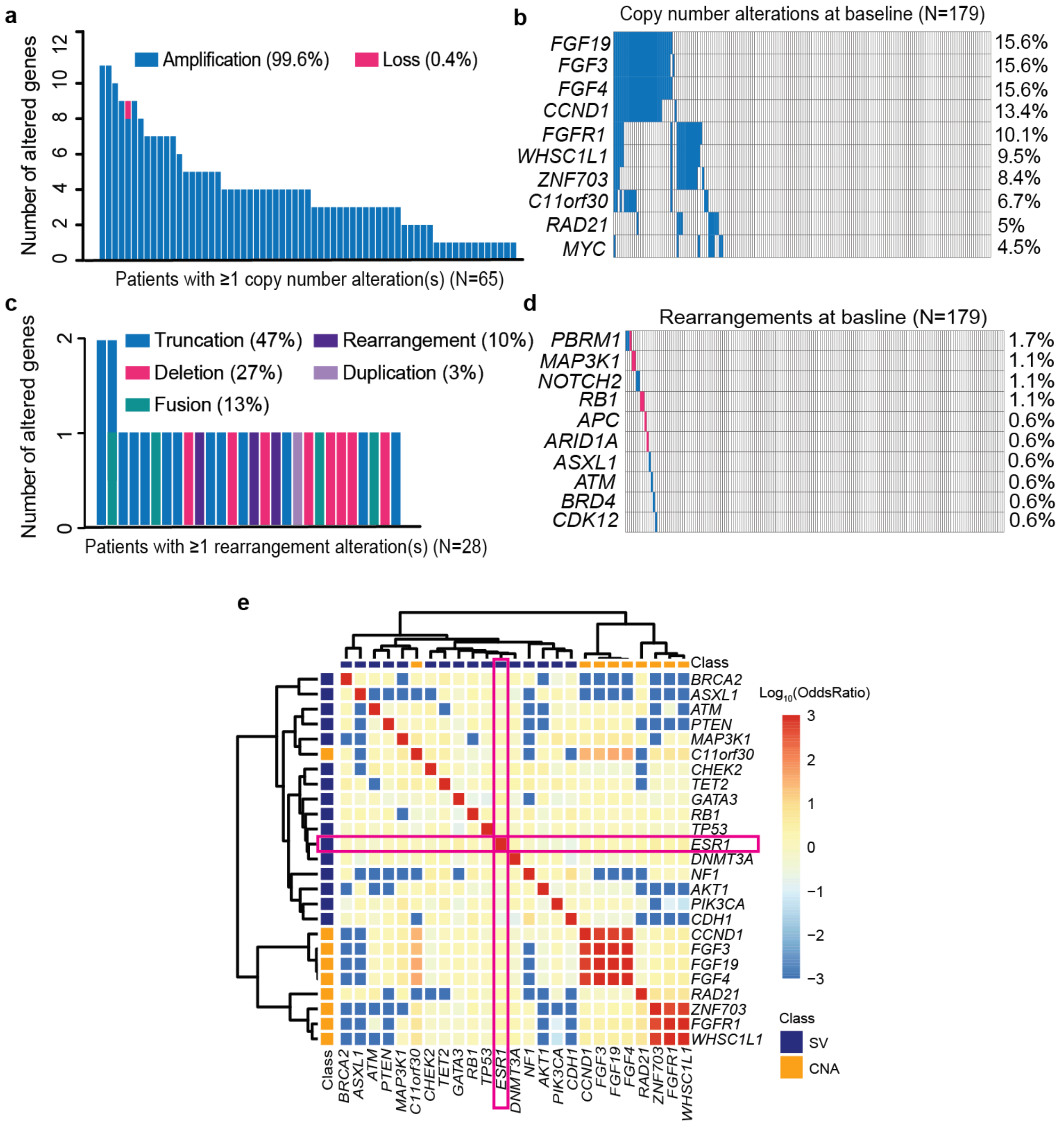

Supplementary Figure 1. Genomic copy number and rearrangement landscape of acelERA 2/3L ER+ aBC patients as measured by liquid biopsy
(**a**) Number of copy number alterations (CNAs) detected per patient and (**b**) prevalence of common pathogenic CNAs. (**c**) Number of rearrangement alterations (RA) detected per patient and (**d**) prevalence of common pathogenic RAs. (**e**) Co-occurrence matrix of short variants (SV)

and CNAs with >5% prevalance, colored by the log_{10}OddsRatio likelihood of co-occurrence within a single patient. SV: short variant, CNA: copy number alteration.

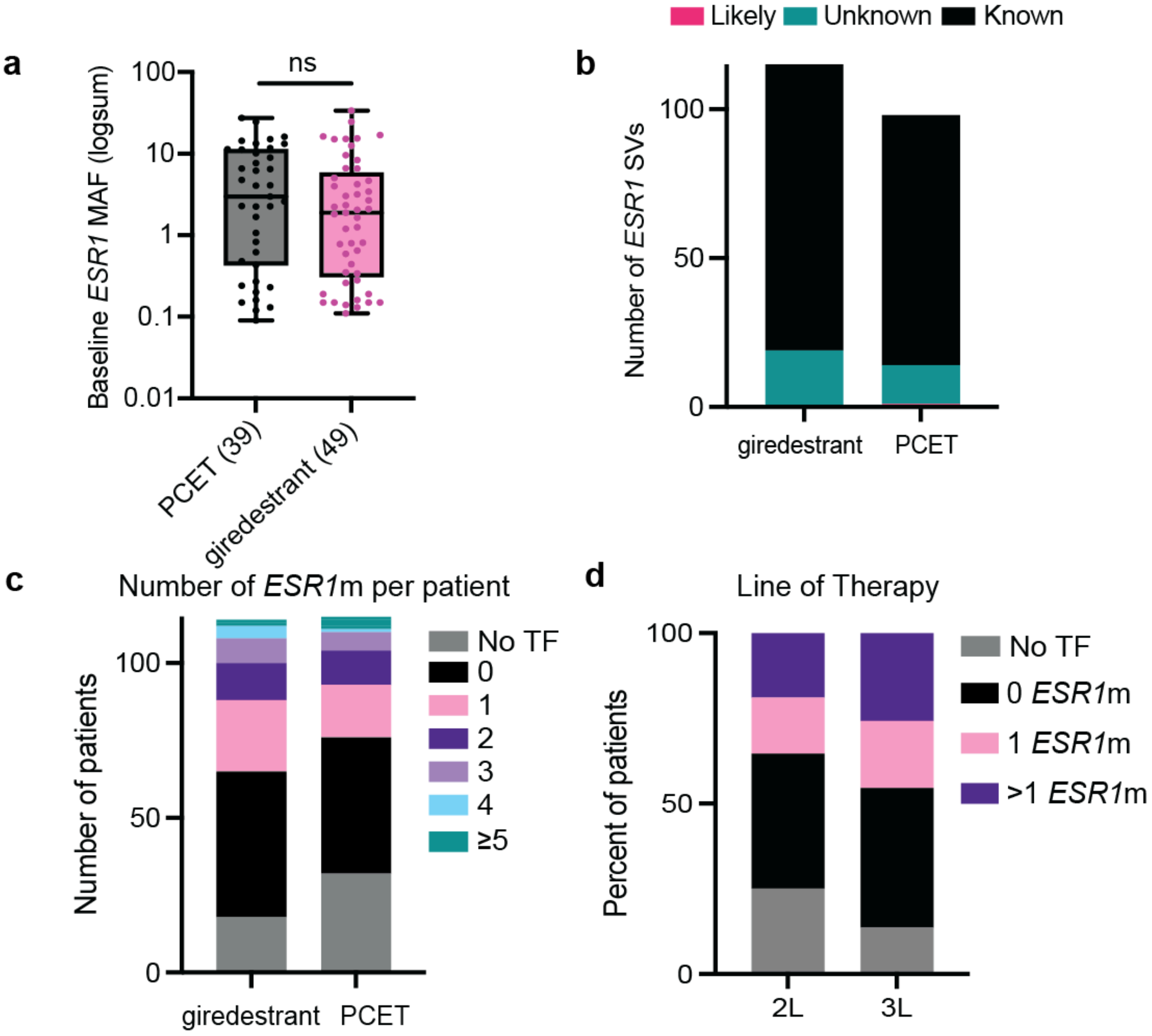

Supplementary Figure 2. Expanded analysis of *ESR1*m characteristics at baseline
(**a**) Baseline *ESR1* mutant allele frequency (MAF) across treatment arms, p=0.3182. (**b**) Prevalence of *ESR1* mutations at baseline, colored by whether the pathogenic significance is likely, known, or unknown. (**c**) Number of *ESR1*m detected per patient, split by treatment arm or (**d**) line of therapy. SV: short variant; PCET: physician's choice endocrine therapy; m: mutation; nmd: no mutation detected; MAF: mutant allele frequency; TF: tumor fraction. Two-sided Mann-Whitney tests were used for group comparisons (p-values indicated); no multiple-comparison adjustment was performed. Source data are provided as a Source Data file.

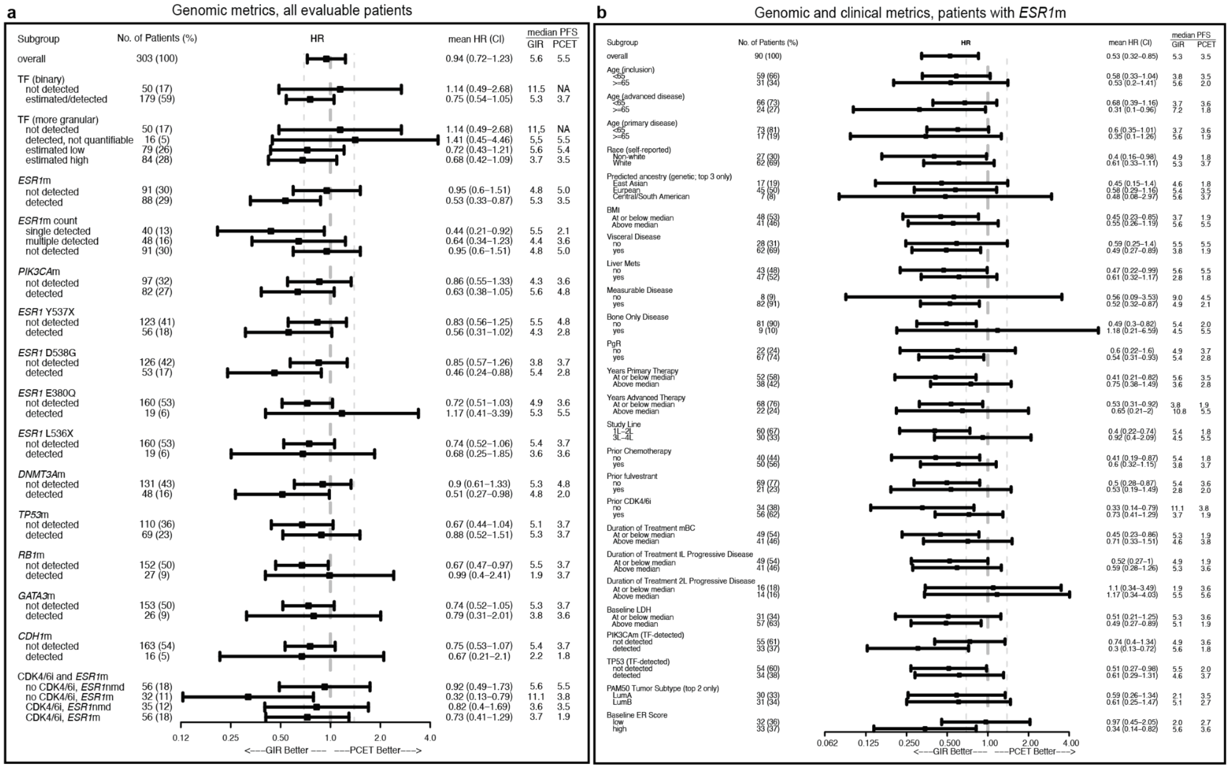

Supplementary Figure 3. ctDNA biomarker and clinical subgroup associations with PFS
(**a**) Forest plot of baseline genomic features associated with progression free survival (PFS) for giredestrant vs. physician's choice endocrine therapy (PCET). TF low or high was defined as below or above the median, respectively. Presence/absence of all alterations was only assessed in the TF-detected subset. "Detected" TF refers to samples with detectable ctDNA that was not quantified. (b) Forest plot of baseline genomic and clinical features associated with PFS, in patients with detectable *ESR1*m. PFS: progression-free survival; m: mutation; nmd: no mutation detected; TF: tumor fraction; PCET: physician's choice endocrine therapy; GIR: giredestrant; PgR: progesterone receptor; BMI: body mass index; LDH: lactate dehydrogenase; HR: hazard ratio; CI: confidence interval. Hazard ratios and 95% confidence intervals were estimated using Cox proportional hazards regression. Source data are provided as a Source Data file.

Subgroup		a both arms No. of Patients (%)	HR	mean HR (CI)	b PCET No. of Patients (%)	HR	mean HR (CI)	c GIR No. of Patients (%)	HR	mean HR (CI)
**TF (binary)**	not detected	50 (22)		Reference	32 (28)		Reference	18 (16)		Reference
	estimated/detected	179 (78)		3.07 (1.96, 4.83)	84 (72)		3.81 (2.09, 6.95)	95 (84)		2.33 (1.16, 4.67)
**TF (more granular)**	not detected	50 (22)		Reference	32 (28)		Reference	18 (16)		Reference
	detected, not quantifiable	16 (7)		2.52 (1.24, 5.10)	7 (6)		2.16 (0.77, 6.10)	9 (8)		2.56 (0.95, 6.90)
	estimated low	79 (34)		2.58 (1.58, 4.21)	37 (32)		3.21 (1.66, 6.18)	42 (37)		1.94 (0.92, 4.10)
	estimated high	84 (37)		3.88 (2.39, 6.28)	40 (34)		5.45 (2.84, 10.46)	44 (39)		2.75 (1.32, 5.72)
***ESR1*m**	not detected	91 (51)		Reference	45 (54)		Reference	46 (48)		Reference
	detected	88 (49)		1.26 (0.90, 1.75)	39 (46)		1.82 (1.12, 2.97)	49 (52)		1.03 (0.65, 1.63)
***ESR1*m count**	single detected	40 (22)		Reference	17 (20)		Reference	23 (24)		Reference
	multiple detected	48 (27)		1.42 (0.88, 2.28)	22 (26)		1.00 (0.50, 1.98)	26 (27)		1.81 (0.94, 3.50)
	not detected	91 (51)		0.95 (0.62, 1.45)	45 (54)		0.55 (0.30, 1.01)	46 (49)		1.30 (0.72, 2.36)
***PIK3CA*m**	not detected	97 (54)		Reference	46 (55)		Reference	51 (54)		Reference
	detected	82 (46)		0.79 (0.57, 1.10)	38 (45)		0.90 (0.56, 1.45)	44 (46)		0.70 (0.44, 1.12)
***ESR1*m Y537X**	not detected	123 (69)		Reference	56 (67)		Reference	67 (71)		Reference
	detected	56 (31)		1.61 (1.13, 2.29)	28 (33)		2.13 (1.26, 3.58)	28 (29)		1.37 (0.83, 2.25)
***ESR1*m D538G**	not detected	126 (70)		Reference	62 (72)		Reference	64 (67)		Reference
	detected	53 (30)		1.16 (0.81, 1.66)	22 (26)		1.65 (0.95, 2.86)	31 (33)		1.04 (0.64, 1.68)
***ESR1*m E380Q**	not detected	160 (89)		Reference	76 (90)		Reference	84 (88)		Reference
	detected	19 (11)		0.86 (0.51, 1.48)	8 (10)		0.64 (0.28, 1.49)	11 (12)		1.08 (0.54, 2.19)
***ESR1*m L536X**	not detected	160 (89)		Reference	76 (90)		Reference	84 (88)		Reference
	detected	19 (11)		1.63 (0.98, 2.71)	8 (10)		1.71 (0.78, 3.79)	11 (12)		1.68 (0.86, 3.30)
***TP53*m**	not detected	110 (61)		Reference	52 (62)		Reference	58 (61)		Reference
	detected	69 (39)		1.02 (0.73, 1.43)	32 (38)		0.91 (0.55, 1.49)	37 (39)		1.18 (0.74, 1.90)
***RB1*m**	not detected	152 (85)		Reference	76 (90)		Reference	76 (80)		Reference
	detected	27 (15)		1.87 (1.20, 2.92)	8 (10)		1.45 (0.66, 3.19)	19 (20)		2.34 (1.34, 4.10)
***GATA3*m**	not detected	153 (85)		Reference	71 (85)		Reference	82 (86)		Reference
	detected	26 (15)		1.10 (0.69, 1.77)	13 (15)		1.02 (0.50, 2.07)	13 (14)		1.16 (0.61, 2.21)
***CDH1*m**	not detected	163 (91)		Reference	77 (92)		Reference	86 (91)		Reference
	detected	16 (9)		1.73 (0.97, 3.06)	7 (8)		2.11 (0.91, 4.92)	9 (9)		1.53 (0.70, 3.35)
**CDK4/6i *ESR1*m**	no CDK4/6i, *ESR1*nmd	56 (31)		Reference	31 (37)		Reference	25 (26)		Reference
	no CDK4/6i, *ESR1*m	32 (18)		0.95 (0.56, 1.59)	13 (15)		1.74 (0.83, 3.62)	19 (20)		0.71 (0.33, 1.53)
	CDK4/6i, *ESR1*nmd	35 (20)		1.74 (1.08, 2.79)	14 (17)		1.85 (0.93, 3.68)	21 (22)		1.91 (0.96, 3.80)
	CDK4/6i, *ESR1*m	56 (31)		2.21 (1.44, 3.38)	26 (31)		2.54 (1.40, 4.63)	30 (32)		2.26 (1.20, 4.26)
			1 2 5			0.5 1 2 5 10			0.5 1 2 5	

Supplementary Figure 4. ctDNA biomarker subgroup association with PFS outcomes across all patients and for each treatment group

(**a**) Forest plot of baseline genomic features associated with progression free survival (PFS) across all patients, or further stratified by treatment with (b) physician's choice endocrine therapy (PCET) or (**c**) giredestrant (GIR). TF low or high was defined as below or above the median, respectively. Presence/absence of all alterations was only assessed in TF-detected subset. "Detected" TF refers to samples with detectable ctDNA that was not quantified. PFS: progression-free survival; m: mutation; nmd: no mutation detected; TF: tumor fraction; PCET: physician's choice endocrine therapy; GIR: giredestrant; PgR: progesterone receptor; BMI: body mass index; LDH: lactate dehydrogenase; HR: hazard ratio; CI: confidence interval. Hazard ratios and 95% confidence intervals were estimated using Cox proportional hazards regression. Source data are provided as a Source Data file.

a

Characteristic	N	TF detected N = 179[1]	TF not detected N = 50[1]	p-value[2]
Treatment	229			0.033
PCET		84 (47%)	32 (64%)	
giredestrant		95 (53%)	18 (36%)	
Age: N (%)	229			0.7
<65		120 (67%)	32 (64%)	
>=65		59 (33%)	18 (36%)	
Race (self-reported)	226			0.005
NON-WHITE		52 (29%)	25 (51%)	
WHITE		125 (71%)	24 (49%)	
Predicted Ancestry (genetic)	174			0.009
African		2 (1.5%)	1 (2.6%)	
Central/South American		13 (9.6%)	0 (0.0%)	
East Asian		35 (26%)	20 (51%)	
European		84 (62%)	18 (46%)	
South Asian		1 (0.7%)	0 (0.0%)	
Postmenopausal	228			0.4
N		22 (12%)	4 (8.0%)	
Y		156 (88%)	46 (92%)	
BMI	226	26.4 (23.5, 30.5)	28.4 (25.3, 32.8)	0.017
Visceral Disease	229	121 (68%)	32 (64%)	0.6
Liver Mets	229	84 (47%)	14 (28%)	0.017
Brain Mets	229			0.4
0		178 (99%)	49 (98%)	
1		1 (0.6%)	1 (2.0%)	
Measureable Disease	229	165 (92%)	47 (94%)	>0.9
Bone Only Disease	229	18 (10%)	6 (12%)	0.7
Non-Measureable, Bone Only	17	11 (79%)	3 (100%)	>0.9
PgR	228			>0.9
N		44 (25%)	12 (24%)	
Y		134 (75%)	38 (76%)	
Years Primary Therapy	228	4.0 (2.0, 8.0)	5.0 (3.0, 9.0)	0.2
Years Advanced Therapy	229	2.00 (1.00, 3.00)	2.00 (1.00, 3.00)	0.4
Study Line	229			0.065
1-2L		123 (69%)	41 (82%)	
3L-4L		56 (31%)	9 (18%)	
Prior Chemo	63	38 (75%)	9 (75%)	>0.9
Prior Fulvestrant	229	37 (21%)	7 (14%)	0.3
Prior CDK4/6i	229	91 (51%)	13 (26%)	0.002
Duration of mBC Therapy (days)	228	660 (419, 1,075)	712 (463, 1,079)	0.6
Baseline LDH	226	238 (204, 338)	199 (179, 224)	<0.001
PAM50 Tumor Subtype	169			0.5
Basal		1 (0.7%)	0 (0%)	
Her2		3 (2.2%)	1 (2.9%)	
LumA		65 (49%)	22 (63%)	
LumB		60 (45%)	12 (34%)	
Normal		5 (3.7%)	0 (0%)	

[1] results displayed as either: N (%) or Median (Q1, Q3)
[2] Pearson's Chi Squared Test, Fisher's Exact Test, Wilcoxon Rank Sum Test

b

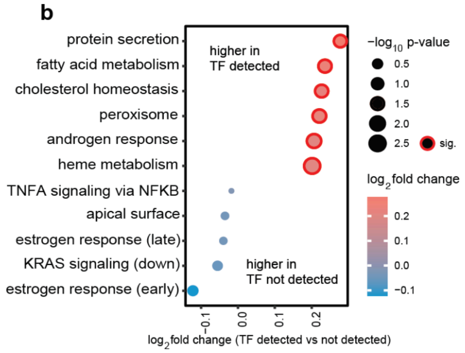

Supplementary Figure 5. Clinical and tumor features associated with ctDNA tumor fraction detection

(**a**) Table of baseline clinical and genomic features associated with ctDNA TF detection. Results are displayed as either N or Median (Q1, Q3). p-values calculated with a Pearson's Chi Squared, Fisher's Exact, or Wilcoxon Rank Sum Test, unadjusted for multiple comparisons. (**b**) RNA-seq gene set variation analysis (GSVA) comparing tumors with vs. without detectable ctDNA at baseline (N=135 detected, N=35 not detected). Top 6 pathways are shown in each direction. N: number of patients; TF: tumor fraction; PCET: physician's choice endocrine therapy; BMI: body mass index; LDH: lactate dehydrogenase; PgR: progesterone receptor. Statistical significance was assessed by fitting a univariate linear model followed by empirical Bayes moderation using the limma package, unadjusted for multiple comparisons. Source data are provided as a Source Data file.

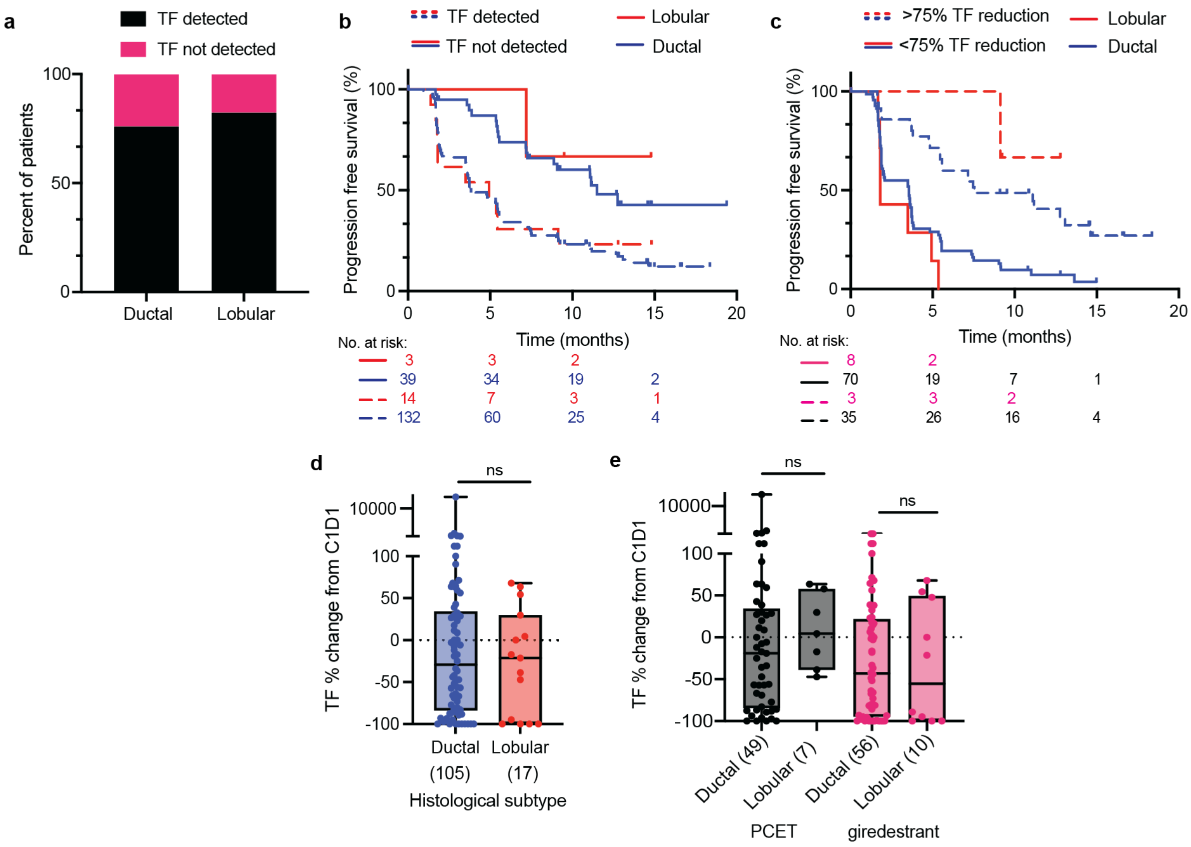

Supplementary Figure 6. ctDNA detection and dynamics are consistent across histological subtypes

(**a**) Percent of patients with detectable ctDNA tumor fraction (TF) by histological subtype. (**b**) Progression free survival (PFS) stratified by TF detection and histological subtype. (**c**) PFS according to histological subtype and baseline TF reduction (>75% at C2D1). (**d**) Percent TF change from baseline to C2D1 split by histological subtype alone (p=0.5736) or (**e**) stratified by treatment arm, p= 0.3140 and 0.6549. PCET: physician's choice endocrine therapy; TF: tumor fraction; PFS: progression-free survival; HR: hazard ratio; CI: confidence interval. Two-sided Mann-Whitney tests were used for group comparisons (p-values indicated); no multiple-comparison adjustment was performed. PFS p-values were determined by two-sided log-rank (Mantel-Cox) tests. Hazard ratios (HR) and 95% confidence intervals (CI) were calculated using the log-rank approach. Source data are provided as a Source Data file.

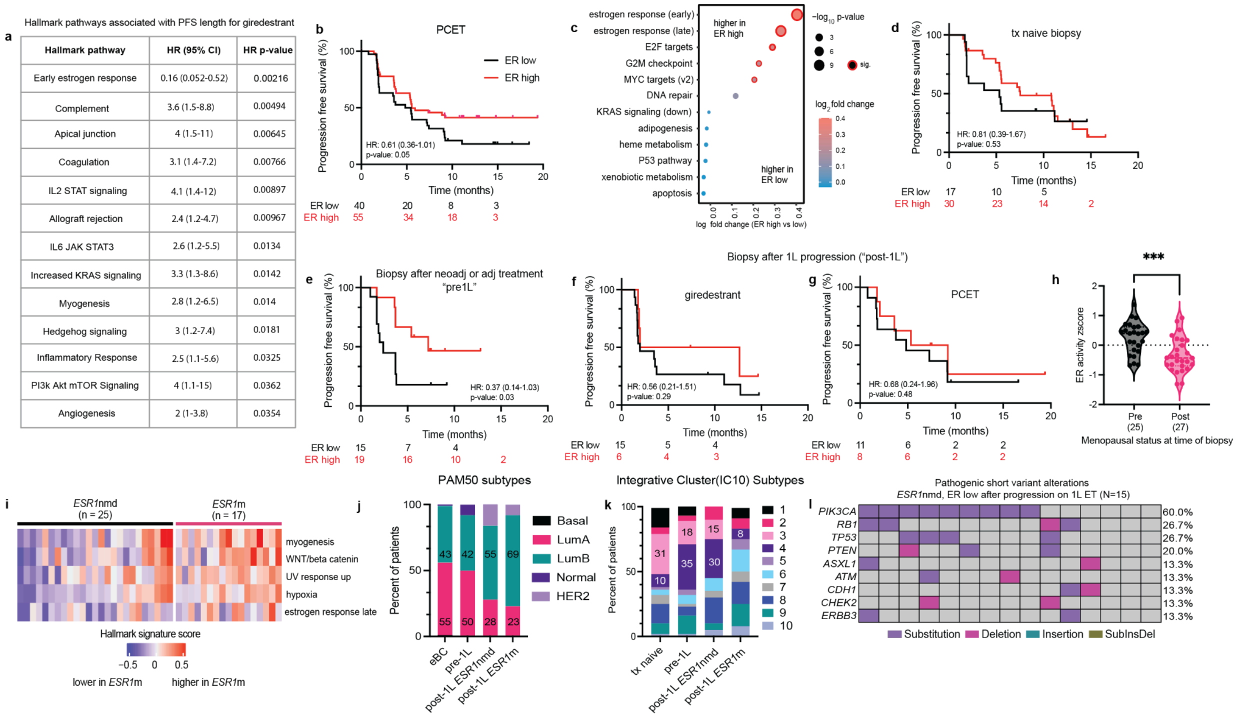

Hallmark pathways associated with PFS length for giredestrant

Hallmark pathway	HR (95% CI)	HR p-value
Early estrogen response	0.16 (0.052-0.52)	0.00216
Complement	3.6 (1.5-8.8)	0.00494
Apical junction	4 (1.5-11)	0.00645
Coagulation	3.1 (1.4-7.2)	0.00766
IL2 STAT signaling	4.1 (1.4-12)	0.00897
Allograft rejection	2.4 (1.2-4.7)	0.00967
IL6 JAK STAT3	2.6 (1.2-5.5)	0.0134
Increased KRAS signaling	3.3 (1.3-8.6)	0.0142
Myogenesis	2.8 (1.2-6.5)	0.014
Hedgehog signaling	3 (1.2-7.4)	0.0181
Inflammatory Response	2.5 (1.1-5.6)	0.0325
PI3k Akt mTOR Signaling	4 (1.1-15)	0.0362
Angiogenesis	2 (1-3.8)	0.0354

Supplementary Figure 7. ER activity is associated with giredestrant PFS outcomes
(**a**) Unsupervised analysis of 50 Hallmark pathways associated with progression-free survival (PFS) in the giredestrant arm; p-value likelihood ratio test, unadjusted for multiple comparisons, (**b**) PFS by tumor ER activity for patients treated with PCET (**c**) RNA-seq GSVA for Hallmark signatures associated with baseline ER activity. (**d**) PFS curves for giredestrant stratified by high (z-score >0) or low (z-score <0) ER activity in naive, (**e**) pre 1L, or (**f**) post-1L biopsies. Post-1L biopsies for the PCET arm are shown in (**g**). (**h**) Tumor ER activity based on patient menopausal status at the time of biopsy, ***p=0.0004. (**i**) Heatmap depicting significantly changed hallmark signaling pathways between *ESR1*nmd and *ESR1*m post-1L tumors. Each column represents a single patient. Blue: downregulated in *ESR1*m, red: upregulated in *ESR1*m. (**j**) Percent of PAM50 or (**k**) IC10 subtype, split by tumor collection timepoint and *ESR1*m status. (**l**) Prevalence of pathogenic short variant alterations in post-1L *ESR1*nmd tumors with high or low ER activity. PFS: progression-free survival; PCET: physician's choice endocrine therapy; tx: treatment; 1L: first line; eBC: early breast cancer; HR: hazard ratio; CI: confidence interval; m: mutation; nmd: no mutation detected; ER: estrogen receptor. RNAseq statistical significance was assessed by fitting a univariate linear model followed by empirical Bayes moderation using the limma package, unadjusted for multiple comparisons. Two-tailed t-tests were used for group comparisons; no multiple-comparison adjustment was performed. Hazard ratios (HR) and 95% confidence intervals (CI) were calculated using the log-rank approach. Source data are provided as a Source Data file.

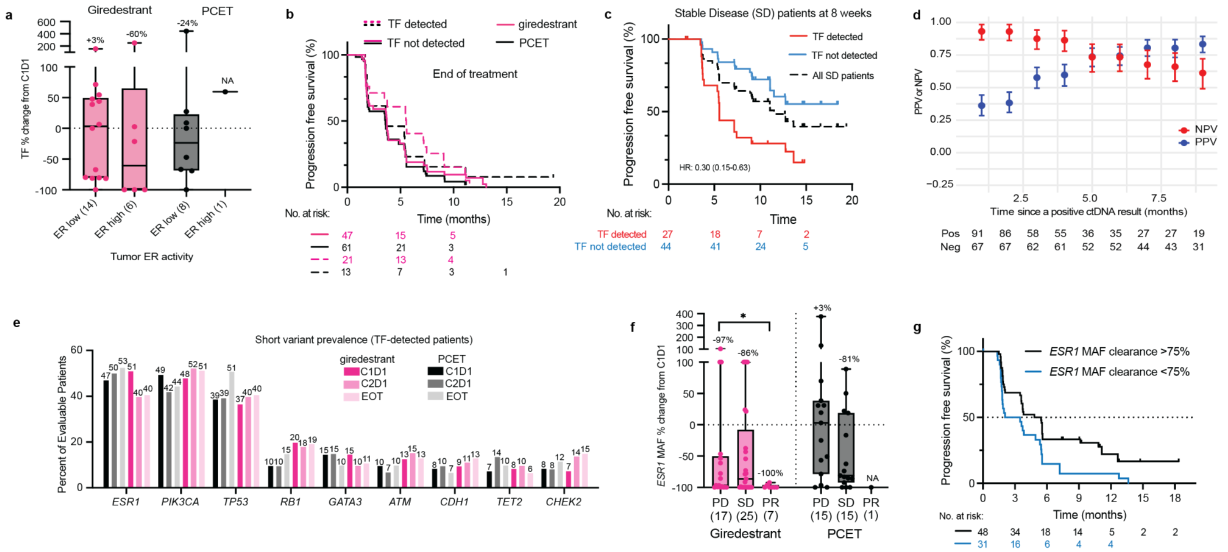

Supplementary Figure 8. Longitudinal ctDNA changes in acelERA
(**a**) Percent tumor fraction (TF) change from baseline to cycle 2 day 1 (C2D1) split by tumor ER activity (split at median). Only samples from post-first line tumors are shown. -100% change indicates that TF is no longer detectable. Median changes are listed above each bar. (**b**) Progression free survival (PFS) according to TF detection at end of treatment (EOT). PCET: physician's choice endocrine therapy. (**c**) PFS for patients with stable disease at 8 weeks, subset by whether TF was detected at C2D1. (**d**) Positive predictive (PPV) and negative predictive (NPV) values for PFS events over different time frames after C2D1. Error bars indicate 95% CIs. (**e**) Prevalence of the most common pathogenic short variants in acelERA split by treatment arm and time point. (**f**) Percent *ESR1* mutant allele frequency (MAF) change from baseline to C2D1 split by treatment arm and confirmed best overall response, *p=0.0401. (**g**) PFS according to whether baseline *ESR1* MAF was reduced by at least 75% at C2D1. PFS: progression-free survival; PCET: physician's choice endocrine therapy; ER: estrogen receptor; TF: tumor fraction; PD: progressive disease; SD: stable disease; PR: partial response; PPV: positive predictive value; NPV: negative predictive value; MAF: mutant allele frequency; C2D1: cycle 2 day 1; EOT: end of treatment. Two-sided Mann-Whitney tests were used for group comparisons (p-values indicated); no multiple-comparison adjustment was performed. PFS p-values were determined with a two-sided log-rank (Mantel-Cox) test and HR and 95% confidence intervals calculated using the log-rank approach. Hazard ratios (HR) and 95% confidence intervals (CI) were calculated using the log-rank approach. Source data are provided as a Source Data file.

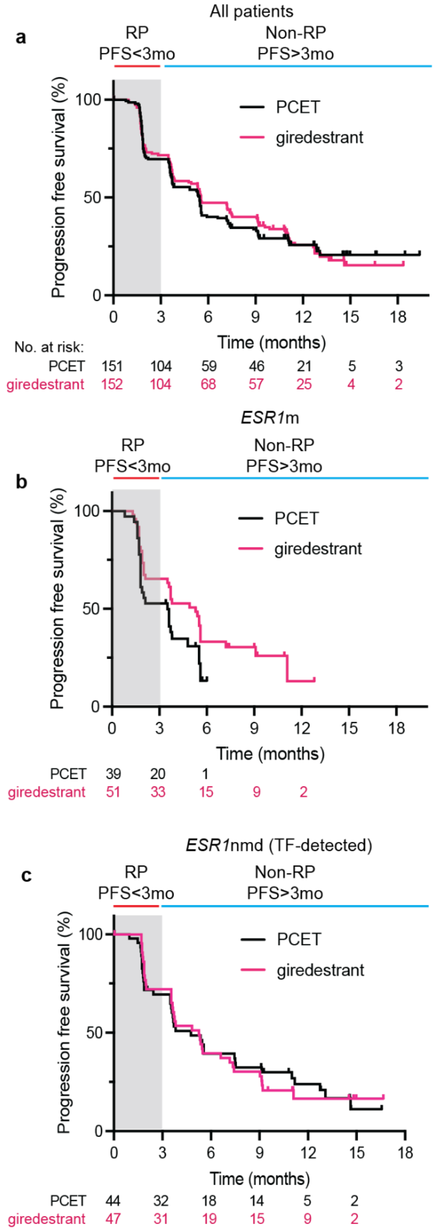

d

Characteristic	N	RP<br>N = 87[1]	non-RP<br>N = 206[1]	p-value[2]
Treatment Arm	293			0.8
PCET		45 (52%)	104 (50%)	
giredestrant		42 (48%)	102 (50%)	
Age	293			0.9
<65		58 (67%)	139 (67%)	
>=65		29 (33%)	67 (33%)	
Race (self-reported)	290			0.7
Non-white		90 (44%)	40 (47%)	
White		114 (56%)	46 (53%)	
Predicted Ancestry (genetic)	168			0.2
African		1 (2.0%)	2 (1.7%)	
Central/South American		5 (10%)	7 (5.9%)	
East Asian		12 (24%)	43 (36%)	
European		31 (62%)	66 (56%)	
South Asian		1 (2.0%)	0 (0%)	
Postmenopausal	292			0.8
N		15 (17%)	34 (17%)	
Y		71 (83%)	172 (83%)	
BMI	290	24.8 (22.5, 27.4)	26.5 (23.6, 31.0)	0.008
Visceral DIsease	293	68 (78%)	133 (65%)	0.022
Liver Mets	293	50 (57%)	64 (31%)	<0.001
Brain Mets	293			0.4
0		84 (97%)	203 (99%)	
1		3 (3.4%)	3 (1.5%)	
Measureable Disease	293	82 (94%)	192 (93%)	0.7
Bone Only	293	6 (6.9%)	20 (9.7%)	0.4
Non-Measureable Bone Only	19	3 (60%)	12 (86%)	0.3
PgR	290			0.11
N		26 (30%)	43 (21%)	
Y		61 (70%)	160 (79%)	
Years Primary Therapy	289	5.0 (3.0, 8.0)	5.0 (2.0, 9.0)	0.7
Years Advanced Therapy	293	1.00 (1.00, 2.00)	2.00 (1.00, 3.00)	0.14
Study Line	293			0.4
1L-2L		59 (68%)	150 (73%)	
3L-4L		28 (32%)	56 (27%)	
Prior Chemo	293	55 (63%)	136 (66%)	0.6
Prior Fulvestrant	293	20 (23%)	36 (17%)	0.3
Prior CDK4/6i	293	52 (60%)	70 (34%)	<0.001
Duration on Therapy mBC (days)	292	598 (417, 840)	709 (457, 1,139)	0.041
Baseline LDH	286	251 (206, 361)	215 (184, 269)	<0.001
Duration of 1L Prior Therapy (days)	292	522 (310, 734)	622 (373, 957)	0.031
Duration of 2L Prior Therapy (days)	84	224 (143, 465)	299 (218, 626)	0.02
PAM50 Tumor Subtype	189			0.2
Basal		0 (0%)	2 (1.5%)	
Her2		2 (3.5%)	3 (2.3%)	
LumA		28 (49%)	69 (52%)	
LumB		23 (40%)	57 (43%)	
Normal		4 (7.0%)	1 (0.8%)	
Tumor ER activity zscore	189	-0.16 (-0.44, 0.14)	0.16 (-0.28, 0.51)	0.001
ctDNA TF	222			<0.001
not detected		4 (6.2%)	45 (29%)	
detected		3 (4.6%)	13 (8.3%)	
estimated		58 (89%)	99 (63%)	

[1] results displayed as either: N (%) or Median (Q1, Q3)
[2] Pearson's Chi Squared Test, Fisher's Exact Test, Wilcoxon Rank Sum Test

Supplementary Figure 9. Clinical and genomic factors associated with rapid progression (**a**) Progression free survival (PFS) according to the treatment arm in acelERA for all patients, patients with (**b**) *ESR1*m, or (**c**) *ESR1*nmd. Interval for rapid progression (RP, 0-3 months) is indicated with a grey box. (**d**) Baseline clinical, tumor, and genomic features associated with rapid progression (RP), which is defined as progression within 3 months of acelERA start. Results are displayed as either N or Median (Q1, Q3). RP: rapid progression; TF: tumor fraction; PFS: progression-free survival; PCET: physician's choice endocrine therapy; m: mutant; nmd: no mutation detected; BMI: body mass index; LDH: lactate dehydrogenase; PgR: progesterone receptor. P-values calculated with either Pearson's Chi Squared, Fisher's Exact, or Wilcoxon Rank Sum Test, unadjusted for multiple comparisons. Source data are provided as a Source Data file.
