# Supplementary material for: ctDNA and tumor-based biomarkers of giredestrant response in acelERA breast cancer
Source: Nat Commun. 2026 Mar 12;17:3848. doi: 10.1038/s41467-026-70335-0 (PMC13121440; doi:10.1038/s41467-026-70335-0)
Supplement: Supplementary file 3 — Reporting Summary [file 41467_2026_70335_MOESM3_ESM.pdf]

nature portfolio

Corresponding author(s): Ann E Collier

Last updated by author(s): Jan 12, 2026

Reporting Summary

Nature Portfolio wishes to improve the reproducibility of the work that we publish. This form provides structure for consistency and transparency in reporting. For further information on Nature Portfolio policies, see our Editorial Policies and the Editorial Policy Checklist.

Statistics

For all statistical analyses, confirm that the following items are present in the figure legend, table legend, main text, or Methods section.

n/a	Confirmed	
☐	☒	The exact sample size (*n*) for each experimental group/condition, given as a discrete number and unit of measurement
☐	☒	A statement on whether measurements were taken from distinct samples or whether the same sample was measured repeatedly
☐	☒	The statistical test(s) used AND whether they are one- or two-sided<br>*Only common tests should be described solely by name; describe more complex techniques in the Methods section.*
☒	☐	A description of all covariates tested
☐	☒	A description of any assumptions or corrections, such as tests of normality and adjustment for multiple comparisons
☐	☒	A full description of the statistical parameters including central tendency (e.g. means) or other basic estimates (e.g. regression coefficient) AND variation (e.g. standard deviation) or associated estimates of uncertainty (e.g. confidence intervals)
☐	☒	For null hypothesis testing, the test statistic (e.g. *F*, *t*, *r*) with confidence intervals, effect sizes, degrees of freedom and *P* value noted<br>*Give P values as exact values whenever suitable.*
☒	☐	For Bayesian analysis, information on the choice of priors and Markov chain Monte Carlo settings
☒	☐	For hierarchical and complex designs, identification of the appropriate level for tests and full reporting of outcomes
☐	☒	Estimates of effect sizes (e.g. Cohen's *d*, Pearson's *r*), indicating how they were calculated

Our web collection on statistics for biologists contains articles on many of the points above.

Software and code

Policy information about availability of computer code

Data collection	No software was used for data collection.
Data analysis	For data analysis, the following software and platforms were used:<br>- Plotting and statistical analysis: GraphPad Prism 10 (v10.1.1)<br>- Bioinformatics: R (v4.2.1 with RStudio)<br>- Publicly available R packages, gene sets, or plotting tools: 'GSNAP' (v 2013-10-10), 'GenomicAlignments', 'limma' (v3.58.1), 'gsva' (v1.50.5), survival' (v3.8-3), 'complexheatmap' (v2.18.0), MSigDB (v2022.1.Hs), 'ggplot2' (v3.5.1), 'boot' (v1.3-31), 'forestmodel' (v0.6.2), 'dplyr' (v1.1.4), 'gmlnet' (v0.6.2), 'forestplot' (v3.1.6),

For manuscripts utilizing custom algorithms or software that are central to the research but not yet described in published literature, software must be made available to editors and reviewers. We strongly encourage code deposition in a community repository (e.g. GitHub). See the Nature Portfolio guidelines for submitting code & software for further information.

Data

Policy information about availability of data

All manuscripts must include a data availability statement. This statement should provide the following information, where applicable:

- Accession codes, unique identifiers, or web links for publicly available datasets
- A description of any restrictions on data availability
- For clinical datasets or third party data, please ensure that the statement adheres to our policy

Source Data are provided with this paper. The Source Data file contains derived, non-identifying data but does not include individual patient-level clinical records. Anonymized, publication-specific biomarker datasets, minimized to those necessary to reproduce the analyses reported here, may be made available upon reasonable request and subject to review and approval by Roche, including execution of a data sharing agreement.
The study protocol is available with the previously published primary analysis (DOI:10.1200/JCO.23.01500). For eligible studies, qualified researchers may request access to individual patient-level clinical trial data underlying the primary study endpoints through a data request platform. At the time of writing, this request platform is Vivli: https://vivli.org/ourmember/roche/. For up-to-date details on Roche's Global Policy on the Sharing of Clinical Information and how to request access to related clinical study documents, see here: https://go.roche.com/data_sharing. Anonymized records for individual patients across more than one data source external to Roche cannot, and should not, be linked due to a potential increase in risk of patient reidentification.

Research involving human participants, their data, or biological material

Policy information about studies with human participants or human data. See also policy information about sex, gender (identity/presentation), and sexual orientation and race, ethnicity and racism.

Reporting on sex and gender	Almost all patients evaluated in this study were female (since this study focused on hormone-receptor positive breast cancer, a disease that primarily affects female patients). These demographics were reported in the primary clinical manuscript (Martin et al. reference #15). Patient sex was determined by an evaluating physician as part of a clinical trial (NCT04576455). Patient gender was not evaluated or collected. Disaggregated sex data is not applicable.
Reporting on race, ethnicity, or other socially relevant groupings	Socially-constructed or relevant categorizations were not used in this study, and were not controlled for.
Population characteristics	Age: Any (majority of patients were age 55 or older)<br>Sex: postmenopausal or pre-/perimenopausal women and men (almost all were female)<br>Genotypic information: Patients were not genotyped as a criteria for enrollment for this clinical trial<br>Current diagnosis: ER+, HER2– locally advanced or metastatic breast cancer<br>Treatment categories: giredestrant or physician's choice endocrine therapy
Recruitment	Recruitment was performed by physicians at individual clinics, per physician's evaluation of eligibility. As described in Martin, et al. (reference #15), key eligibility criteria were: "postmenopausal or pre-/perimenopausal women, and men, age 18 years or older who had received one or two prior lines of systemic therapy for ER1,HER2– locally advanced or metastatic BC. One of the prior lines must have been ET for ≥6months; ≤1 targeted agent (including, but not limited to, CDK4/6is) was allowed; and one of the lines may have included chemotherapy. Patients had measurable (per RECIST v1.119) or bone-only disease, which must have had ≥1 predominantly lytic bone lesion. Key exclusion criteria included prior treatment with an investigational SERD (prior fulvestrant was allowed, as long as it was terminated ≥28 days before random assignment); advanced, symptomatic, visceral spread that risked life-threatening complications in the short term; known active uncontrolled or symptomatic CNS metastases, carcinomatous meningitis, or leptomeningeal disease; and active cardiac disease or history of cardiac dysfunction."
Ethics oversight	As described in Martin, et al., acelERA BC "was designed by the senior academic authors and representatives of the sponsor (F. Hoffmann-La Roche Ltd, Basel, Switzerland). Data were collected by the sponsor and analyzed in collaboration with the senior academic authors, who vouched for the completeness and accuracy of the data and analyses, and for the fidelity of the study to the protocol. acelERA BC was performed in accordance with Good Clinical Practice guidelines and the Declaration of Helsinki. Protocol approval was obtained from an independent ethics committee for each participating site. Every patient gave written informed consent. An internal monitoring committee comprising employees of the sponsor reviewed cumulative safety data periodically throughout."

Note that full information on the approval of the study protocol must also be provided in the manuscript.

Field-specific reporting

Please select the one below that is the best fit for your research. If you are not sure, read the appropriate sections before making your selection.

[x] Life sciences   [ ] Behavioural & social sciences   [ ] Ecological, evolutionary & environmental sciences

For a reference copy of the document with all sections, see nature.com/documents/nr-reporting-summary-flat.pdf

Life sciences study design

All studies must disclose on these points even when the disclosure is negative.

Sample size	As described in Martin et al., "The planned sample size was 300 patients. The primary analysis was planned for when approximately 166 INV-PFS events from both arms had occurred; this enabled 80% power to detect a target INV-PFS hazard ratio (HR) of 0.647 (corresponding to animprovementinmedianINV-PFSfrom 5.5 to approximately 8.5 months) at a 5% (two-sided) level of significance. The largest HR determined to be statistically significant (minimal detectable difference) was approximately 0.738."
Data exclusions	ctDNA data (other than ESR1 mutation status) from patients with PredcineCARE results were excluded from most analyses in this manusscript. This assay did not provide tumor fraction calculation and is not directly comparable to the F1LCDx results.
Replication	Not applicable for clinical data. Technical replicates were not performed for ctDNA or tumor RNAseq due to limited sample material. Standard QC procedures, as recommended by the manufacturers, were followed for all assays.
Randomization	As described in Martin et al. "Eligible patients were randomly assigned 1:1 using a permuted-block method to receive giredestrant or PCET. Random assignment was stratified by disease site (visceral [lung and/or liver involvement] v nonvisceral), prior CDK4/6i (yes v no), and prior fulvestrant."<br><br>For the biomarker analysis of the acelERA trial, the allocation of samples into experimental groups was dictated by the randomized design of the parent clinical study and the subsequent availability of evaluable biological material. Since this was a retrospective analysis of a randomized controlled trial, many baseline covariates were balanced by the initial randomization. However, to ensure the robustness of the biomarker findings, additional controls were implemented. In the differential gene expression and GSVA analyses, the limma package utilized a linear modeling framework that accounts for variability across the dataset. Key clinical covariates, such as prior CDK4/6i therapy duration and the presence of visceral metastases were used as stratification factors or included in multivariate models to determine the independent predictive value of the identified biomarkers. Only samples meeting strict quality control thresholds for RNA integrity and ctDNA concentration were included in the final analysis to minimize technical bias.
Blinding	acelERA BC was a Phase II, randomized, open-label, multicenter study. As described in Martin et al. "All radiologic data (eg, computed tomography scan, magnetic resonance imaging, bone scan), photographs of skin lesions,and any additional clinical information required were sent to a blinded, independent, core imaging laboratory (contracted by the sponsor) to facilitate a retrospective evaluation of disease response and progression for the full population by a blinded independent review committee (BIRC)."<br><br>Investigators were not blinded to treatment allocation or clinical outcomes during the biomarker analysis. This approach was necessary because the study's primary objective was to correlate specific molecular features (such as ESR1 mutation status and ER transcriptional activity) with observed clinical responses and progression-free survival to identify predictive signatures. Furthermore, the computational and statistical analyses of tissue RNA-seq and liquid biopsy data were performed according to a predefined bioinformatics pipeline, which utilizes objective, standardized algorithms (e.g., GSVA and limma) that are inherently resistant to investigator bias.

Reporting for specific materials, systems and methods

We require information from authors about some types of materials, experimental systems and methods used in many studies. Here, indicate whether each material, system or method listed is relevant to your study. If you are not sure if a list item applies to your research, read the appropriate section before selecting a response.

Materials & experimental systems

n/a	Involved in the study
☒	☐ Antibodies
☒	☐ Eukaryotic cell lines
☒	☐ Palaeontology and archaeology
☒	☐ Animals and other organisms
☐	☒ Clinical data
☒	☐ Dual use research of concern
☒	☐ Plants

Methods

n/a	Involved in the study
☒	☐ ChIP-seq
☒	☐ Flow cytometry
☒	☐ MRI-based neuroimaging

Clinical data

Policy information about clinical studies

All manuscripts should comply with the ICMJE guidelines for publication of clinical research and a completed CONSORT checklist must be included with all submissions.

Clinical trial registration	NCT04576455
Study protocol	The clinical study protocol can be accessed via the primary clinical manuscript (Martin et al. reference #15) and/or via this link: chrome-extension://efaidnbmnnnibpcajpcglclefindmkaj/https://ascopubs.org/action/downloadSupplement?doi=10.1200%2FJCO.23.01500&file=protocol_JCO.23.01500.pdf

Data collection	As describein Martin et al., "Between November 27, 2020, and October 27, 2021, 303 patients were enrolled (151 to giredestrant, 152 to PCET) across 85 sites in 17 countries. At clinical cutoff (February 18, 2022; median follow-up: 7.9months), 106 patients (35.0%) were on treatment, 257 (84.8%) remained on study, and 46 (15.2%) had discontinued the study."
Outcomes	As described in Martin et al. "The primary analysis was planned for when approximately 166 INV-PFS events from both arms had occurred; this enabled 80% power to detect a target INV-PFS hazard ratio (HR) of 0.647 (corresponding to an improvement in median INV-PFSfrom 5.5 to approximately 8.5 months) at a 5% (two-sided) level of significance. The largest HR determined to be statistically significant (minimal detectable difference) was approximately 0.738. Data for patients without disease progression ordeathatclinicalcutoffwerecensoredatthetimeofthelast tumor assessment (or at the time of random assignment 11 day if no tumor assessment was performed after the baseline visit). INV-PFS was compared between arms using the stratified log-rank test, with the HR estimated using a stratified Cox proportional hazards model (stratified by disease site, priorCDK4/6i, and prior fulvestrant). Unstratified analyses were used for subgroups (excluding the secondary endpoint of INV-PFS in ESR1m tumors). For each treatment arm, Kaplan-Meier methodology was used to estimate median INV-PFS, and the Brookmeyer-Crowley method was used to construct the 95%CIs. OS was to be hierarchically tested using the same methodology if the primary endpoint was statistically significant at the primary analysis."

Plants

Seed stocks	*Report on the source of all seed stocks or other plant material used. If applicable, state the seed stock centre and catalogue number. If plant specimens were collected from the field, describe the collection location, date and sampling procedures.*
Novel plant genotypes	*Describe the methods by which all novel plant genotypes were produced. This includes those generated by transgenic approaches, gene editing, chemical/radiation-based mutagenesis and hybridization. For transgenic lines, describe the transformation method, the number of independent lines analyzed and the generation upon which experiments were performed. For gene-edited lines, describe the editor used, the endogenous sequence targeted for editing, the targeting guide RNA sequence (if applicable) and how the editor was applied.*
Authentication	*Describe any authentication procedures for each seed stock used or novel genotype generated. Describe any experiments used to assess the effect of a mutation and, where applicable, how potential secondary effects (e.g. second site T-DNA insertions, mosiacism, off-target gene editing) were examined.*
